# Professional Character

**Published:** 1858-06

**Authors:** F. S. Slosson


### PROFESSIONAL CHARACTER.

BY DR. F. S. SLOSSON.

*An Address, delivered on retiring from the chair, at the late meeting of the Dental Convention of Northern Ohio.*

FRIENDS AND BRETHREN OF THE PROFESSION—We welcome you to our city again.

At each succeeding convention, our stand-point will be raised, and the limits of our vision extended.

As we look back over years of toil, and trial, and doubt, we see much at first view that we could wish obscured from sight and from memory; but every trial, and every failure too, has done something to elevate us to our present high position.

When we look around us, we wonder that so much could have been done with such material, in so short a time; and, as we extend the spinal column to its greatest tension, and poise on the tip of our toes to peer into the future, we see light and glorious success ahead.

As we look over the profession through its periodicals, we think we have some little reason for self-congratulations, as we see those standing high in our esteem just beginning to admit the correctness of our treatment of teeth which we have practiced for years, with so much success as to warrant the hope that the time is not far distant when the object of pursuit will be fully attained. I mean the treatment of alveolor abscesses the plugging over exposed nerves, and filling from the apex of the root, &c.

But, as these topics will be discussed during the progress of this Convention, I propose to speak on the subject of professional character.

That our profession should have a character, as distinctly marked as that of law, medicine or divinity, will not be questioned.

Character is the representations of a man as to personal qualities. These may be associated, as well as individual character.

What is it that distinguishes one man from another but the representation of different personal qualities?

What is it but character that gives authority to the words, and importance to the decisions of one man above his fellows?

Why is it that we attach more importance to the opinions of one person, than we do to those of another? In another's language, character indictates the degree in which a man possesses creative spiritual energy, is, in short, the only expression of the man, the person.

If this is its true definition, and I think it is, it shows that we are personally responsible for the cultivation of those qualities which will elevate us as individuals, and thus elevate the profession at large. Actions are the manifestations of character. Persistence is that quality that does most to establish it.

The man who together possesses knowledge, energy and skill, accompanied with high moral qualities, is the man who will do most for the profession. He possesses true dignity, has little of selfishness, and is above those jealousies and bickerings which disturb little minds. He is accessible to all; and his opinions are anxiously sought, and as freely given; he secures his own, by seeking the public good. His design is, that all he does shall be well done; and if he fails, as sometimes the best will do, he is ready to make it good. His charges are neither too high nor too low, as either extreme will destroy confidence. In short he is governed by the higher law of doing as he would be done by.

How strongly in contrast with such a man is that ignoramus whose advent into a town or city is heralded by a shower of cards, bills, and advertisements, found in every vehicle in our streets, in every yard, and under every door, proclaiming the thousands of teeth set and filled during the last few weeks, inviting the community to improve this opportunity, as they may never see his like again! Such a one deals in base metals, soft solder, soft soap, and has a *softer* head.

His patients pay a higher price in proportion to the value received, although he charges but "two and sixpence" for plugs, and a bit or a picayune for extracting.

He, being a bit of a picayune man, the perfume of his soap is exceedingly attractive with a certain portion of community whose ideas assimilate his own.

Between these two men we have every shade and grade of character. We are happy to know that the number who approximate the first are daily increasing; yet it is lamentably true that our profession is dogged in its upward progress, by a multitude who assimulate the latter.

I said that actions are the manifestations of character. It is not one or two great, good or noble acts that make the character of a man. It is formed only by numberless little acts.

As dust shows the way the wind blows, so do the little acts of a man indicate more truly his real character than great ones. One little act does but little to establish a man's character, yet it does a great deal to destroy it.

How quickly do we form an opinion of a man from his advertisements; and the reason is that they are so unlike those of other professions.

We are prone to get upon a hobby of some kind; and the motive power is in the personal pronoun; for instance: "*I* extract teeth with a peculiar kind of instrument; *I* plug over or destroy the nerve without pain; *I* plug with crystal, gold or succedaneum; *I* make porcelain plate, or porcelain teeth; and *I* have the very latest patent for everything relating to the profession;" to all of which is added the recommendation of some professor, clergyman or surgeon.

Any such resort shows the man to be wanting in one essential quality, of a good professional character, viz: selfrespect, in as much as his design is to get from his brethren, in this low way, that which he might have by high and honorable procedure.

All of this savors of quackery; and the longer and more numerous the recommendations, the greater the humbug. The fact is, that if we are riding a hobby of any kind, it will be very likely to appear in our advertisements with head and tail up.

Character is the embodiment of the acts of a man. I said persistence is that quality that does most to establish it.

It is the stream that has longest run that has made the deepest cut in the mountain rock; so is it with this quality. It is the persistance, the tenacity, which holds a man to his work, that makes the strongest mark upon him. How often do we see men of most brilliant minds and fertile imaginations fail to make or establish a character that does not expire with their breath, and for this reason, that they do not possess this quality to any considerable extent. While, on the other hand, we see men of moderate attainments, and less brilliant minds, engraving their names and their characters on a nation's heart, and in a nation's history.

Persistency in a good enterprise, inspires confidence. It shows the man in his true character, that he is tenacious of his principles, and of his profession. While his opposite is all things to all men, and nobody to every one.

This quality is more effective for evil than for good, because of the downward tendencies of our natures. It is persistence in an evil course that has furnished the victims for our prisons and the scaffold, and is forcing, yearly, thousands of intemperate men down to a drunkard's grave.

I have dealt the more on this quality, because of its importance, and the results which might be accomplished if each individual would persistently pursue such a course as would fit him for his work and his profession.

Let any young man determine that on the next tooth, he will spend all the time necessary to excavate and prepare it for the reception of the gold, then introduce slowly, and condense thoroughly, till there is left not the slightest place for the point of a sharp instrument, then burnish and polish, until he can say, I have done the best I am able; and thus proceed with the next, and the next, persistently holding to his determination; will it be long ere he will stand in the front ranks of the profession in this department?

There are three things necessary to the attainment of character:

An object to be aimed at; the means by which it is to be attained, and persistence in the use of those means. If a young man aims to be first in his profession, his object is a noble one, and is attainable if he improve his time and the means within his reach, (and they are a thousand fold more now than when some of us commenced,) if he tenaciously holds to his determination and to his work, until it becomes the object of his highest aspiration. As another has said: "The true merchant, the true statesman, the true military commander, becomes a man of character only when he puts on and identifies himself with his particular profession or art."

This shows us why many accomplish so little, and rise no higher in the profession. It also shows why so much deception is practiced; such as extracting teeth that might have been plugged, plugging some cavities that are accessible, and leaving others that are not; taking out plugs which are preserving the teeth, for the sake of replacing them; putting in plugs of gold that are two thirds tin; putting in plates of gold that are all silver but the gold deposited on them by galvanism.

These are little things, but they are things we have often seen, and they as strongly indicate character as larger ones.

This is taking a dollar and cent view of the whole practice. The aim is low and the practice is lower.

When a patient takes our chair he commits to us a personal trust, not dollars and cents, but of a trust infinately more sacred; and any person who does not feel its responsibility and is recreant of the trust, is unfit for his position, and unworthy of a standing in our profession.

But, brethren, we are persuaded better things of you, though we thus speak.

No one has attained eminence in his calling, but by long years of toil, trial and embarrasments, in the primary principles of his profession. Every step in his progress has been attained with unforeseen difficulties, which he has mastered; and thus, step by step, has he followed his own aspirations to the summit of human glory. While another one, surrounded by circumstances equally, or more advantageous, whose aim is low, has hardly a respectable standing in his profession, as a writer in the "Atlantic," speaking of this kind of person, says: "If they are in the learned professions, they are tricksters in law, quacks in medicine, formalists in divinity, though regular practitioners in all, and clients will be cheated, and patients will be poisoned, and parishioners will be—we dare not say what, though all the colleges in the universe had showered on them their diplomas."

Now, why is this? The one relies on his own native energies, improving every means in his power, learning something from every one; while the other relies more on his connections, or the circumstance which surrounded him, or some title of D. D., M. D., or D. D. S., all of which are good, and gratifying, and always retiring when worthily bestowed; but these are his advertisements, and his capital, and they are the true index of his character.

It is said of Daniel Webster that, on receiving his diploma, he retired, and in the presence of his class-mates, tore it in pieces. Whether this is true or not, it is eminently true that he relied entirely on his own native energy; that he laid, with his hands, every stone in the foundation on which was to be built the character of that illustrious statesmen.

The dental profession is in its infancy. The members of it have been for years like so many men deep down in some dark mine, each one working, and carrying his light on his own head, borrowing none and reflecting none. But when they meet, as sometimes they do, on such occasions as the present, the light is concentrated, dispelling the gloom of that narrow-minded selfishness which dries up and shuts up all the better and nobler qualities of the soul. The veriest sprig in the profession feels its influence, and straightens up himself to be more of a man, while some of the older ones, who feel they are not the only objects of God's special favor, the reservoir of all his choicest blessings, the depository of all the wisdom and intelligence in the land.

But this is a day of light, of action and of progress—the mind should be enlightened by the steady, light of intelligence, by which all our actions should be guided. We ought not to be so occupied with the present, that we cannot act in reference to the future. I rejoice to see in our profession an elevating power, a germ of progress, and of those principles which are to place it in a high position for usefulness.

The dental colleges and periodicals, the conventions, in which are the free interchange of views of practice, are facilities for improvement, which a few years since we knew nothing of.

It makes us wish to obliterate the past. But, as without the past, there would be no future, and as every elevation must have some means of ascent, we will rejoice if the past be made the stepping-stone by which any shall rise to higher attainments, or to a more lofty eminence.

Brethren, in leaving the chair, I thank you for the honor conferred in placing me in it, and I hope the same harmony and good feeling which has pervaded the convention over which it has been my honor to preside, may characterize the one now in session.

